# Beyond the Bench: Healthy Home, Healthy Community

**DOI:** 10.1289/ehp.115-a194

**Published:** 2007-04

**Authors:** Tanya Tillett

We like to think of home as a safe haven, but sometimes this shelter can harbor a wide range of health hazards. Children living in urban, low-income, and minority neighborhoods are often at greater risk of exposure to home-based health hazards than other groups, but implementing affordable, effective methods to alleviate the risk can be challenging. A new interactive museum housed in a residential property in Rochester, New York, now offers children and adults hands-on, real-world examples of hidden hazards in the home along with guidelines on simple preventive measures.

For more than two years, the Environmental Health Sciences Center (EHSC) of the University of Rochester and its community partners, the Southwest Area Neighborhood Association (SWAN) and the Rochester Fatherhood Resource Initiative (RFRI), worked together to develop an easily accessible, community-based program that would promote environmental health in the home. In June 2006 they launched the Rochester Healthy Home museum.

Eleanor Coleman, SWAN’s community asset manager, explains, “We have found that community residents respond better to hands-on education than to printed materials.” Katrina Korfmacher, the center’s Community Outreach and Education Core coordinator, adds, “Our role was to bring together the existing resources within the community, develop the technical information for displays, and support the initial setup of the Healthy Home.”

Korfmacher says the center and its community partners spent several months locating a residential building that was large enough to accommodate groups of visitors, solving commercial zoning issues, and finding a landlord who was amenable to such a unique project. The Healthy Home is located adjacent to the historic Susan B. Anthony neighborhood in a community with high poverty, a low number of owner-occupants, and one of the highest lead poisoning rates in the city.

The neighborhood location is important to SWAN outreach coordinator Shehrina Tabassum, who says, “We wanted it to be accessible to low-income residents who might not be able to travel across town to visit a Healthy Home.” According to a 2002 report by the Center for Governmental Research, 34% of the children living in the SouthWest neighborhood represented by SWAN have blood lead concentrations above the CDC “threshold of concern” of 10 μg/dL. James Richardson, president of a residents’ group called the Lennox Street Block Club, notes that many of the century-old homes in this area have environmental hazards and that nearby residents will benefit from the interactive displays at the Healthy Home. “When it is hands-on, people seem to believe it a little more,” he says.

The partners focused on some of the major risks present in many homes—asthma triggers, lead hazards, household chemicals such as pesticides and cleaners, and indoor air quality hazards—to create topical posters and hands-on displays. For example, the “Lead Room” has three display windows that demonstrate different methods for treating painted window frames for lead hazards, including instructions and cost information for lead-safe work practices to minimize the disturbance of lead-based paint. The “Asthma Safe Bedroom” displays pillowcase covers and other asthma trigger reduction tools, and offers tips for avoiding mold growth along with other appropriate housekeeping guidelines. In the kitchen, visitors find displays explaining the risks of exposure to certain common household chemicals as well as tips for safe handling and storage. The Healthy Home also educates visitors about potential causes of injuries such as electrical shock and choking hazards, as well as possible carbon monoxide poisoning risks such as defective furnaces.

With emphasis on the idea that creating a healthy home environment is a shared responsibility, the partnership encourages visits from all community stakeholders. In its first six months of operation, the Healthy Home has welcomed more than 400 visitors, including residents, students of all ages, teachers, contractors, property owners, and elected officials, all of whom can apply the lessons learned in the museum to their respective community roles. The Healthy Home hosts outreach activities such as support group meetings (for example, for families of asthmatic children), neighborhood block group meetings, and free safe practices training courses (by the end of 2006, 67 people had received training in a series of lead safety courses). The partnership also conducts community presentations and distributes information at health fairs on the topics covered in the Healthy Home.

Admission to the Healthy Home is free, and guided tours are available. In the near future, the partners plan to increase outreach efforts through youth programs, home health visiting nurses, and others. Meanwhile, center staff are evaluating the impact of the Healthy Home on visitors’ knowledge and behaviors, and are writing a guide to help other communities develop similar facilities.

**For More Information** http://www2.envmed.rochester.edu/envmed/ehsc/outreach/CommunityPartners/CommunityPartnersHH.html

## Figures and Tables

**Figure:**
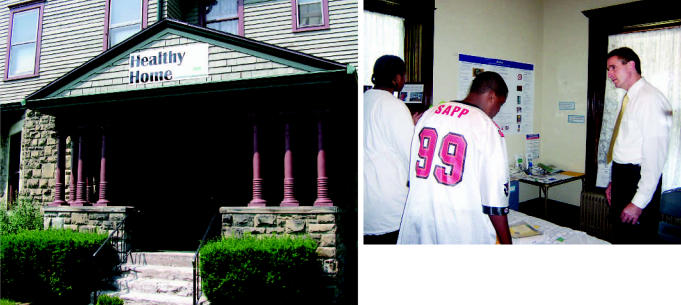
Musings on a healthy environment The Healthy Home interactive museum teaches community members how to identify and mitigate health hazards in residences. (Above) SWAN high school interns Scott Blue and Isiah Johnson teach Rochester mayor Robert Duffy about healthy housekeeping in the “Asthma Safe Bedroom.”

